# Absolute and relative pitch: Global versus local processing of chords

**DOI:** 10.2478/v10053-008-0152-7

**Published:** 2014-02-20

**Authors:** Naomi Ziv, Shulamit Radin

**Affiliations:** 1 Department of Psychology, College of Management Academic Studies, Israel; 2 Department of Behavioral Sciences, Tel Aviv-Yafo Academic College, Israel

**Keywords:** absolute pitch, relative pitch, global processing, local processing

## Abstract

Absolute pitch (AP) is the ability to identify or produce notes without any reference note. An ongoing debate exists regarding the benefits or disadvantages of AP in processing music. One of the main issues in this context is whether the categorical perception of pitch in AP possessors may interfere in processing tasks requiring relative pitch (RP). Previous studies, focusing mainly on melodic and interval perception, have obtained inconsistent results. The aim of the present study was to examine the effect of AP and RP separately, using isolated chords. Seventy-three musicians were categorized into four groups of high and low AP and RP, and were tested on two tasks: identifying chord types (Task 1), and identifying a single note within a chord (Task 2). A main effect of RP on Task 1 and an interaction between AP and RP in reaction times were found. On Task 2 main effects of AP and RP, and an interaction were found, with highest performance in participants with both high AP and RP. Results suggest that AP and RP should be regarded as two different abilities, and that AP may slow down reaction times for tasks requiring global processing.

## Introduction

Absolute pitch (AP) is the ability to identify and name isolated tones without comparison to any reference pitch (Miyazaki, 2004a). Although traditionally assumed to be a rare “all or none” ability, scholars today believe this ability is continuous, with many individuals showing various degrees of AP (e.g., Vitouch, 2003). While most individuals encode music in terms of relations between pitches of successive notes, AP possessors perceive music in terms of the absolute pitch of the constituting notes, and treat isolated pitches categorically (Schlaug, 2001; Schulze, Gaab, & Schlaug, 2009; Schulze, Mueller, & Koelsch, 2013). This tendency develops in early childhood, related to the acquisition of language (Deutsch, Henthorn, Marvin, & Xu, 2006; Pfordresher & Brown, 2009), and dependent both on innate predisposition and learning experience (Baharloo, Service, Risch, Gitschier, & Freimer, 2000; Gregersen, Kowalsky, Kohn, & Marvin, 2000; Theusch & Gitschier, 2011; Zatorre, 2003). Although AP has long been considered a unique gift in musicians, allowing faster and more accurate perception of certain musical features, there is an ongoing debate today regarding its advantages. The main issue in this context is whether the perception of isolated pitches may not interfere with processing of global aspects of music, for which relative pitch (RP) is more appropriate. The aim of the present study was to examine this question in relation to the perception of chords.

Music has a hierarchical organization, and its perception requires both local processing (e.g., in perceiving specific pitches or intervals and the duration of sounds) and global processing (such as in perceiving general contour; see Warren, 2008). In normal individuals, perceptual global processing seems to precede local processing (Peretz, 1990; Schiavetto, Cortese, & Alain, 1999; Stewart, Overath, Warren, Foxton, & Griffiths, 2008). Contour information, for example, is remembered after short-time intervals, whereas pitch (specific interval) information requires long-term memory (Dowling & Bartlett, 1981). Since the most important musical aspects are constructed on pitch *relations* and not individual pitches (Miyazaki, 2004a), certain authors maintain that RP is an important ability for music perception, whereas AP is irrelevant (Miyazaki, 2004a; Miyazaki & Rakowski, 2002; Ward, 1999). However, others maintain that AP is indeed relevant to music, and leads to superior performance on various tasks (Dooley & Deutsch, 2010, 2011).

In recent years, differences between AP possessors and non-possessors have been explored in brain studies. Numerous studies have shown differences between AP possessors and non-possessors in brain structure. For example, an asymmetry in the planum temporale, an area involved in the abstraction of properties of complex sounds, has been found, with smaller right planum temporale in AP possessors, suggesting the influence of early exposure as well as innate factors (Keenan, Thangaraj, Halpern, & Schlaug, 2001; Schlaug, Jancke, Huang, Staiger, & Steinmetz, 1995; Wilson, Lusher, Wan, Dudgeon, & Reutens, 2009). Similarly, differences in brain activity have been found (a) in processing in tasks such as identifying melodic intervals, with AP possessors showing smaller P3 amplitudes and shorter latencies than non-possessors (Hantz, Crummer, Wayman, Walton, & Frisina, 1992); (b) in tone labeling, with a bias in AP possessors towards the left hemisphere, a bias towards the right hemisphere in non-AP possessors (Brancucci, di Nuzzo, & Tomassi, 2009), and generally more brain activity in both hemispheres in AP possessors than non-AP possessors (Wilson, Lusher, Wan, Dudgeon, & Reutens, 2009; Wu, Kirk, Hamm, & Lim, 2008); and (c) memory for pitch, with more activation in the left superior temporal sulcus in AP possessors in the early encoding phase (Schulze et al., 2009).

As for the performance of AP possessors and non-possessors on various tasks, results seem inconsistent. Several studies found that on certain tasks, AP possessors’ performance is reduced, whereas non-AP possessors’ performance remains uninfluenced. Mito (2003) asked participants to play a melody by sight-reading, either on a normal or on a transposed keyboard. Whereas non-possessors showed no significant differences in performance between the two keyboards, a significant decline in AP possessors’ performance was found in the transposed keyboard. Similarly, Miyazaki (2004b), and Miyazaki and Rakowski (2002) found that AP possessors’ identification of transposed melodies which were aurally or visually presented was reduced, and reaction times were longer, whereas non possessors were not affected by transposition. However, Dooley and Deutsch (2010, 2011) found better performance in AP possessors than non-possessors in musical dictation and in identifying intervals between two successively presented pitches. Finally, others found no differences between AP possessors and non-possessors in the identification of tonic and mode of presented randomly generated melodies in major and minor mode and in reaction times (Temperley & West Marvin, 2008), or in the identification of melodic intervals (Benguerel & Westdal, 1991).

Some of the inconsistent results may be explained by the dichotomous classification of experimental groups to AP possessors and non-possessors. In fact, AP is not a yes/no capacity. Rather, it is regarded as a continuum (Bahr, Christensen, & Bahr, 2005; Krumhansl, 2000; Takeuchi & Hulse, 1993; Vitouch, 2003). Indeed, performance within AP possessors on various tasks, such as accuracy and speed of pitch identification, is influenced by factors such as timbre, key color (white or black) range, and tonality (Bahr et al., 2005; Miyazaki, 1990; Takeuchi & Hulse, 1993; Vanzella & Schellenberg, 2010). Likewise, AP possessors vary in their abilities to perform RP tasks (Benninger, Granot, & Donchin, 2003). Moreover, in Miyazaki and Rakowski’s (2002) study, mentioned above, the variance in performance within AP possessors in the identification of transposed melodies was greater than that of non-AP possessors.

Levitin and Rogers (2005) suggest a continuum on which AP is at one extreme and RP at the other. However, even in studies which divided AP into three levels, results are not unequivocal. Wilson and colleagues (2009) divided 36 musicians into AP, quasi-AP (QAP), and RP groups by their results on single pitch identification. Participants who identified 90% or more pitches correctly were categorized as AP, those who identified 20% or less as RP, and those with intermediate results as QAP. Participants were then tested on two tasks, one requiring AP, and the other requiring RP. In both tasks, an arpeggiated chord was presented. In the AP task, participants were asked to name the final note, and in the RP task, they were asked to decide whether or not a tone presented following the chord was the tonic. Their analysis focused on differences between AP and QAP. In the AP task, AP possessors showed more accuracy, followed by QAP (though there were no significant differences in reaction time) and RP possessors. In the RP task, QAP musicians showed significantly faster mean reaction time for correct tonal classification compared with correct pitch naming, whereas the reverse was true for AP musicians. In other words, possessing AP may slow down processes for which RP is more appropriate. Temperley and West Marvin (2008) asked 30 musicians, divided into three levels of AP in a similar manner, to identify the tonic and the mode of presented melodies. In their study, no significant differences were found between the groups either in the identification of tonic and mode or in reaction times. Finally, Dooley and Deutsch (2010) divided 60 participants into these three groups and tested them on musical dictation. They found significant differences between the groups, with AP possessors showing the best performance, followed by borderline possessors and non-possessors.

The seemingly contrasting results suggest two things. First, the different tasks may require different strategies. Indeed, in analyzing the results obtained in various studies, several authors suggest difference sin processing modes to explain either the variance within AP possessors, or the differences in performance between AP possessors and non-possessors. Thus, Miyazaki and Rakowski (2002) suggest that the automatic nature of AP may interfere with RP judgments in AP possessors, accounting for the longer reaction times in identifying transposed melodies. Similarly, Wilson and colleagues (2009) mention that several AP musicians spontaneously reported mentally translating tones from their pitch names to their RP classification, thus explaining the slower mean reaction time of AP musicians for correct tonal classification compared with QAP musicians. Mito (2003) suggests that AP possessors have weak RP and rely only on AP, leading to their reduced performance of playing by sight-reading on a transposed keyboard. Terhardt and Seewann (1983) conclude that whereas both AP and non-AP possessors rely on pitch in determining key, AP possessors base their decision on the identification of individual pitches, whereas non-possessors deduce a feeling of key from a series of notes. Finally, Benguerel and Westdal (1991) maintain that in identifying sequential intervals, both AP and non-AP possessors use RP.

Second, these findings may raise doubts regarding the validity of conceiving AP and RP as extremes on the same continuum. According to Miyazaki (2004a), AP and RP are different modes of musical pitch processing, having incompatible features. If AP and RP tasks require different strategies, perhaps the two should be regarded as separate continuous abilities. Thus, an individual could be high or low on each, and their ability to use the different strategies on various tasks, requiring AP or RP, would depend on their specific ability level on each continuum. Indeed, it is curious that in studies in which the tasks are explicitly defined by the authors as requiring RP (Mito, 2003; Miyazaki & Rakowski, 2002; Temperley & West Marvin, 2008; Wilson et al., 2009), only AP ability was used as a measure to differentiate participants. In the present study, both AP and RP abilities were measured separately, and a classification of participants by their performance on both was used to categorize them into groups.

Of the studies conducted on AP, only very few examined perception of isolated chords (McDermott & Oxenham, 2008). Chords, which are simultaneous combinations of three or more pitches, present an interesting case for examining AP in the context of global and local processing. On the one hand they constitute a single object, but at the same time they are composed of discrete pitches, organized in pre-established intervals. The notes comprising a chord may coalesce into musical Gestalts providing harmonic information (Heaton, 2003), or may be perceived as simultaneously presented pitches (McDermott & Oxenham, 2008).

To our knowledge, Wilson and colleagues’ (2009) study was the only one to examine isolated chords in relation to AP. However, they did not directly investigate the perception of whole chords. Evidence from several other studies, which did not look at AP, seems to suggest that chords tend to be perceived as a whole unit. In a study on tonal fusion, DeWitt and Crowder (1987) found in a sample of non-musicians that combinations of two or three pitches which could be interpreted as deriving from partials based on a fundamental frequency were harder and slower to classify as multiple tones than tones which could not be interpreted that way. Since in chords the frequencies of the composing tones often share several partials, this may suggest that chords should tend to fuse and are not necessarily perceived as a sum of their components (McDermott & Oxenham, 2008). Platt, Racine, Stark, and Weiser (1990) compared the performance of musicians (defined as having at least 3 years of musical training) and non-musicians on the adjustment of a comparison tone to a specific note in an A major chord presented in either root position or in first inversion. Such a task requires analytical perception of the chord’s pitches, in order to match the note to a particular pitch within the chord. Musically untrained participants had more difficulty ignoring the component notes than musicians, suggesting a global perception of the chord pitches. Likewise, Demany and Ramos (2005, Study 1) had participants with musical background listen to random (inharmonic) chords of pure tones and asked them to judge whether a pure tone presented was identical to one of the chord’s notes. They found participants had difficulty in this task, and concluded that chord components were hard to perceive individually. Finally, in Parncutt and Bregman’s (2000) Study 1, musicians and non-musicians heard chords (major, minor, diminished) followed by a probe-tone, and had to decide whether the probe-tone was similar to the preceding chord. Whereas musicians gave higher ratings to notes belonging to the chords than to notes which were not part of the chord, non-musicians’ ratings of notes did not differ, suggesting the perception of chords in non-musicians as one unit, and an inability to analyze their components.

The aim of the present study was to examine the relative effect of AP and RP abilities on the perception of isolated chords. Although related, based on the literature above, we hypothesized the two abilities would affect AP and RP tasks differently. Two tasks were used, requiring RP and AP. In the RP task, participants were asked to identify the chord quality (major/minor/augmented/diminished), a skill which is traditionally learned in solfeggio exercises (musical hearing and singing exercises) and music theory classes, whereas in the AP task, they were asked to identify a single pitch within the chord. In light of the reviewed literature, several hypotheses were formulated:

1. High RP would lead to more accurate chord quality identification than AP.

2. High AP would lead to more accurate pitch identification within a chord than RP.

3. High AP would slow down responses on the chord quality task.

4. High AP would quicken reaction times on the pitch identification task.

## Method

### Participants

Seventy-three participants took part in the study (*M*_age_ = 25.67, **SD** = 4.42), 52 males and 21 females. Participants were all practicing musicians specializing in various domains (performance, composition, conducting, musicology studies) with high levels of formation in hearing and solfeggio. Mean age of starting music lessons was 9.19 years (*SD* = 4.05). Mean number of years of studying music was 13.61 (*SD* = 4.58), 5.07 years of studying solfeggio (*SD* = 3.36) and 6.16 years of studying theory (*SD* = 3.45). Participants took part in the study voluntarily.

### Materials

#### Demographic questionnaire

Participants filled out a questionnaire containing items regarding age, gender, and music education.

#### Tasks

Four tasks were designed for the study, two pre-test tasks, designed to classify participants by AP and RP, and two experimental tasks, using a computer program designed for the study. The sound chosen for the tasks was a regular piano sound by the computer synthesizer.

##### Pre-test 1: without interference

**Figure 1.**
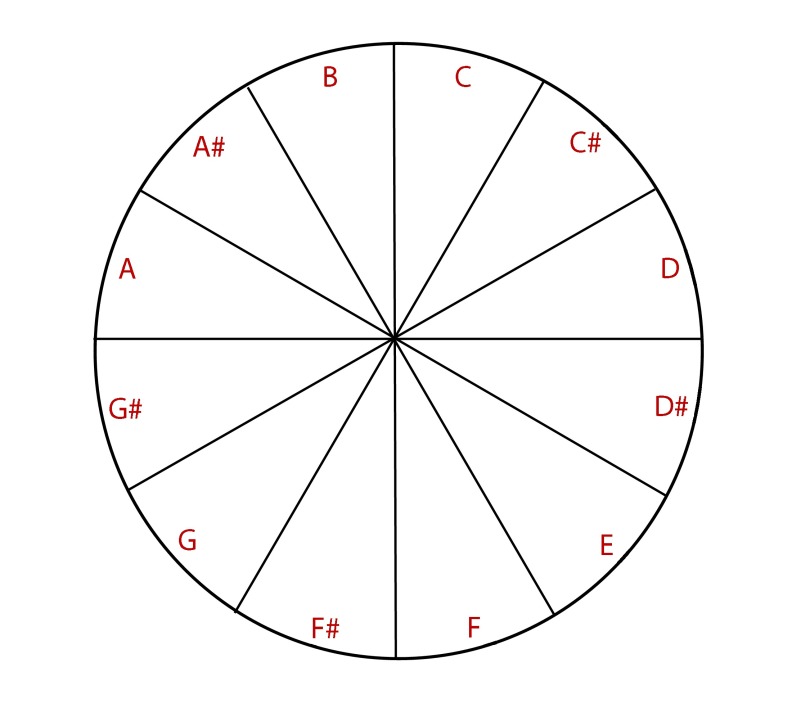
Presentation of notes.

Each test consisted of five training trials, followed by 15 test trials. At first, a message on the screen appeared, explaining the task and informing the participant that the training phase is about to start. When the participant was ready to begin, he/she pressed the left mouse button. A blank screen then appeared for 1 s, after which a circle appeared with the curser positioned in its middle. The circle was divided into 12 sections representing all seminotes in an ascending order clock-wise (see Figure 1). After 1 s, a piano note was heard for 1 s. The participant had to identify the note as quickly as possible by positioning the mouse on the presented note on the screen and pressing the left button on the mouse. Once the participant chose a note (or if he/she failed to answer within 8 s), the screen changed into a blank screen for 1 s, followed by the circle again and the following tone, with the curser repositioned at the center of the circle. After five training trials, a written message appeared on the screen, informing the participant that the training phase is over and the test will begin once he/she presses the mouse key. The test phase was identical to the training phase and consisted of 15 trials. In many studies (e.g., Bahr et al., 2005; Dooley & Deutsch, 2011; Wilson et al., 2009), measuring AP is done with tones ranging several octaves, with all successive notes from different octaves, in order to disturb the use of RP. In the present study, since the aim of the pre-test was to infer RP, as will be explained below, the notes used were all 12 notes from the same octave. The program recorded each presented note as well as the note chosen by the participant as a number. Then each reply was coded by the program as right (1) or wrong (0). Order of presented notes was random for each participant.

Scores were calculated for the test phase alone. For each participant, a total AP score was calculated as the sum of correctly identified notes. A correct reply was given a score of 1, and a wrong reply a score of 0. In addition, an RP score was calculated as the interval between each two consecutive notes; for example, if C was the first note presented, followed by D, the interval equaled 2 (semi-tones). The interval between each two consecutive answers was compared to the parallel interval between correct replies. If the interval was identical, a score of 1 was given, if it was different, the score was 0. For example, if the correct reply between C and D was 2, and the participant identified D and E, he/she would also receive a 2, that is, it would be considered a correct interval. The aim of this calculation was to test the participants reply in reference to him/herself. That way, each note was actually scored in reference to the preceding note only. It should be noted, however, that the RP score in this case was not directly measured, but inferred by the identification of intervals between successive pitches. The correct identification of intervals between two successive pitches has been used in previous studies to examine RP (e.g., Dooley & Deutsch, 2011; Foster & Zatorre, 2010; Zatorre, Perry, Becket, Westbury, & Evans, 1998). Measuring RP is indeed difficult in the case of AP possessors, since even in judging intervals, they may use AP to determine the pitch of one tone, then the other, and calculate the difference. RP possessors, on the other hand, would deduce the interval without categorizing each pitch separately. In the present case, it is impossible to know which strategy was used by participants, but conceivably, a participant using only RP, could achieve a perfect score. A total RP score was calculated by adding all scores on each pair of trials.

##### Pre-test 2: with interference

The second pretest was identical to the first, including a training phase, with one difference - after the participant chose a note and prior to hearing the next note, a rapid sequence of random notes was played for 4 s. Order of presented notes was randomized for each participant. The aim of the interference was to avoid basing each consecutive reply on the previous reply, thus relying on RP. Since in Pre-test 1, participants basing their replies on RP could conceivably receive perfect AP scores, the interference in Pre-test 2 would differentiate between AP possessors and RP possessors.

AP and RP scores were calculated in the same way as in Pre-test 1.

##### Test 1

**Figure 2.**
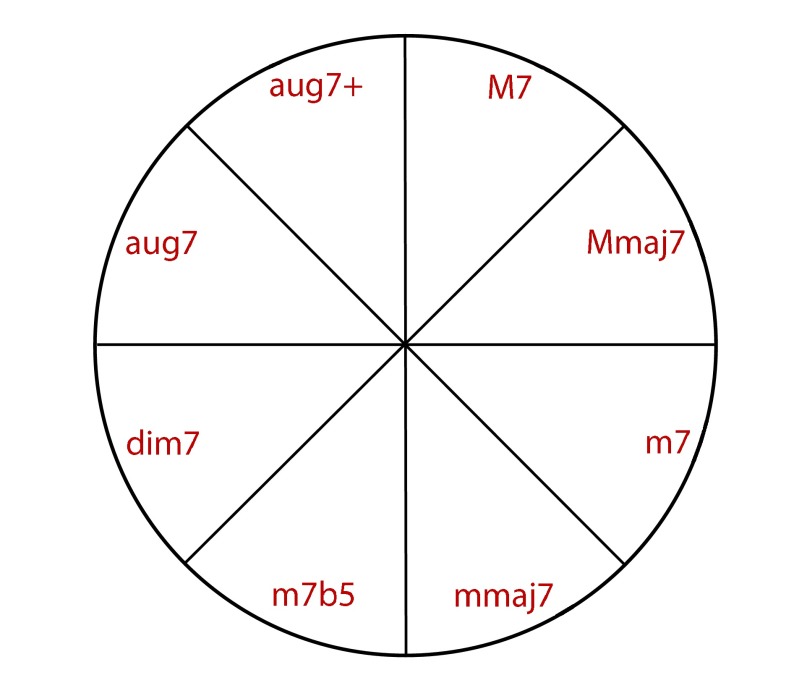
Representation of chord types.

As in the pre-tests, in each test five training trials preceded the 15 test trials. First, a screen appeared explaining the task and asking the participant to press the left mouse button once he/she is ready to start the training phase. A blank screen was then presented for 1 s, followed by a circle representing chord types (see Figure 2). In each trial, a random 7th chord was played for 2 s, in root position or second inversion. Seventh chords were used since simple triads were thought to be too simple a task for musicians. The participant was asked to choose as quickly as possible the type or harmonic hue of the chord by positioning the curser on the chosen chord from the circle and pressing the left button of the mouse (if the participant did not answer after 8 s, the next chord was played). The following types of 7th chords were used: dominant 7th, major 7th, minor 7th, minor major 7th, minor 7thbth, augmented 7th, and augmented major 7th. The chords were constructed on random notes in all scales. Presentation order of chords was randomized for each participant.

A score was calculated by summing the correct replies on all 15 trials. In addition, a mean reaction time score was calculated for all trials.

##### Test 2

A blank screen was shown for 1 s, followed by a screen on which the words “C major” were written, and the chord C major was played for 1 s. The reason a reference chord was given was to provide non-AP possessors with a reference stimulus, in order to be able to perform the task. It was assumed that for non-AP possessors the lack of a reference tone would render the task too difficult. After a 150-ms pause, the screen changed to show the circle of notes presented in the pre-tests, with a question presented above the circle: “What is the X note from the bass in the next chord?” (Questions were randomly asked about the first, second, and third tones from the bass, i.e., the second, third, and fourth notes of the chord, respectively.) After 2 s a chord was played for 2 s. The participant had 15 s to place the curser on the chosen note and press the left button before the screen changed to a blank screen again, followed by the reference chord (C), and the next chord. The longer time interval given for replies in this test was chosen because of the relative difficulty of the task. The same chords as in Test 1 were used, in root position, or second inversion. Order of presented chords was randomized for each participant.

A total score was calculated by summing the correct replies on all 15 trials. In addition, a mean reaction time for all trials was calculated.

### Procedure

Each participant arrived at a time fixed by phone and met the researcher in a quiet room at the school of music at Tel-Aviv or Jerusalem University. The researcher gave each participant a debriefing page explaining the aims and procedure of the experiment. Once the participant read the text they were seated in front of a portable computer with headphones. The researcher explained the study was about musical perception and the exact tasks would appear on the screen. The researcher added that if there were any problems in understanding the task the participant could call the researcher and ask for help. She then asked the participant to put on the headphones and start the program. The researcher then exited the room and waited outside. After the first two pre-tests the participant called the researcher, who came in and asked the participant to leave the room and wait outside for a few minutes. After a few minutes break, the researcher asked the participant to go back to the room and continue with the two tests. The researcher set the program and left the room again. After the study the participant was asked to call the researcher back. The researcher went into the room, asked the participant to fill out the demographic questionnaire, and thanked him. Each procedure lasted about 15-20 min.

## Results

### Pre-tests

Mean scores on AP and RP for Pre-tests 1 and 2 are presented in Table 1. Correlations between scores ranged from .608 (between RP scores on Pre-test 1 and AP scores on Pre-test 2) to .805 (between RP and AP scores on Pre-test 2). All correlations were significant at *p* < .001. These high correlations suggest that the two abilities are strongly related. Correlations were run between results on these tests and age of starting music education. A negative correlation was found between AP scores on Pre-test 2 and age of starting music education (*r* = -.336, *p* = .004), confirming previous studies indicating a relationship between starting age of musical training and AP (Deutsch et al., 2006; Takeuchi & Hulse, 1993).

**Table 1.** Mean Scores for AP and RP Pre-tests 1 and 2

	AP Pre-test 1	RP Pre-test 1	AP Pre-test 2	RP Pre-test 2
Mean	7.45	7.91	6.41	6.47
	(5.65)	(4.24)	(5.12)	(4.12)
Median	8	8	6	6

*Note.* Standard deviations in parentheses.

Pre-tests 1 and 2 were designed to differentiate between AP and RP, through the presence or absence of interference. Participants were divided by two criteria, according to median scores. First, they were divided by AP so that participants scoring 6 (the median on Pre-test 2) or higher on AP in Pre-test 2 (since in Pre-test 1 there was no interference, participants with AP would necessarily receive a high score on RP as well) were categorized as *high AP*, and the others as *low AP*. Second, they were divided by RP so that participants with an RP score of 8 (the median on Pre-test 1) and higher in Pre-test 1 only (since Pre-test 2 contained interference, replies could not be based on RP, by remembering the preceding note) were defined as *high RP*, whereas those who scored lower than 8 were defined as *low RP*. It should be noted, however, that the category of “high AP” is nominal and does not necessarily imply high AP. Indeed, participants who scored 6 on Pre-test 2 do not have high AP, but simply have higher scores than participants who scored less than 6. Using this classification, 28 participants were categorized as high AP/high RP (22 males, six females), nine participants as high AP/low RP (four males, five females), nine participants as low AP/high RP (eight males, one female), and 27 as low AP/low RP (18 males and nine females). Since, as mentioned above, RP and AP are highly correlated, the number of participants in groups high on one ability and low on the other is significantly smaller than in groups either high or low on both. Means and standard deviations of scores of the four categories are presented in Table 2. It should be emphasized that AP and RP are both considered continuous abilities, and the division of participants into separate groups of high and low AP and RP was done in order to create groups differing on their position on these continuums.

**Table 2.** Means of AP and RP Scores on Pre-tests 1 and 2

	hAP/hRP	hAP/lRP	lAP/hRP	lAP/lRP
Pre-test 1	11.92	4.22	10.33	4.18
RP scores	(2.2)	(2.27)	(2.23)	(1.75)
Pre-test 2	11.89	7.44	1.66	1.96
AP scores	(2.75)	(1.33)	(1.65)	(1.55)

*Note.* Standard deviations in parentheses. hAP/hRP = high AP-high RP; hAP/lRP = high AP-low RP; lAP/hRP = low AP-high RP; lAP/lRP = low AP-low RP.

In order to confirm the categorizing of participants into high and low AP and RP, the effectiveness of the interference in Pre-test 2 in distinguishing between high and low AP and RP was tested by paired-sample *t*-tests. These were first conducted separately for participants who were categorized as high and low AP, between AP scores on Pre-test 1 and 2. The idea was that if a participant possesses high AP, the interference in Pre-test 2 should not affect the score and it would be identical to that in Pre-test 1 (without interference). If some participants do not possess high AP, they may still score high on Pre-test 1, basing their replies on RP. However, the interference in Pre-test 2 would render this strategy impossible, and so their score on this test should be lower. Then, comparisons were conducted separately for participants who were categorized as high and low RP, between RP scores on Pre-test 1 and 2. Here, participants basing their replies on RP who received high scores in Pre-test 1 should be affected by the interference in Pre-test 2, and receive a lower score on Pre-test 2. Participants with low RP should not be affected by the interference in Pre-test 2, and receive equally low scores on both pre-tests. Results are shown in Table 3. Using the Bonferroni correction, alpha was set at .0125. As can be seen, participants who were categorized as low AP did indeed score lower on Pre-test 2, with the interference. Thus, the interference task was efficient. Likewise, participants with high RP were affected by the interference in Pre-test 2, and their performance was reduced, whereas participants with low RP were not affected by the interference, and their performance was equally low in both pre-tests. These results confirm that the pre-tests successfully distinguished between AP and RP.

**Table 3.** *t*-Tests of High and Low AP Participants Petween Scores on Pre-tests 1 and 2

	Pre-test 1 mean	Pre-test 2 mean	*t*
High	11.43	10.81	1.037
AP-AP scores	(4.46)	(3.13)	
Low	3.55	1.88	2.77*
AP-AP scores	(3.59)	(1.56)	
High	11.54	9.21	4.1*
RP-RP scores	(2.29)	(3.57)	
Low	4.19	3.66	1.06
RP-RP scores	(1.86)	(2.41)	

*Note.* Standard deviations in parentheses. *p < .01.

One-way ANOVAs were conducted between participants categorized as high and low AP, and between participants categorized as high and low RP on age of starting music lessons and on number of years of practicing and studying music. In line with previous studies (Deutsch et al., 2006; Takeuchi & Hulse, 1993), a significant difference was found between high and low AP possessors on age of starting music lessons, *F*(1, 72) = 5.33, *p* = .024. High AP possessors’ mean age of starting music lessons was lower (*M* = 8.14, *SD* = 3.31) than that of low AP possessors (*M* = 10.27, *SD* = 4.49). No significant differences were found between high and low RP possessors.

### Tests 1 and 2

As mentioned above, the task on Test 1 was the identification of chord hues. The task on Test 2 was the identification of specific pitches within a chord. A maximum score would be 15. For the whole sample, mean score on Test 1 was 7.11 (*SD* = 4.13) and for Test 2 it was 4.89 (*SD* = 2.98). The difference between the two was significant (*t* = 4.5, *p* < .001). Mean reaction time for Test 1 was 4836.82 ms (*SD* = 1198.43) and for Test 2 it was 6591.76 ms (*SD* = 1966.38). The difference here was also significant (t = -6.87, *p* < .001). This indicates that beyond AP and RP abilities, Test 2 was more difficult.

**Figure 3.**
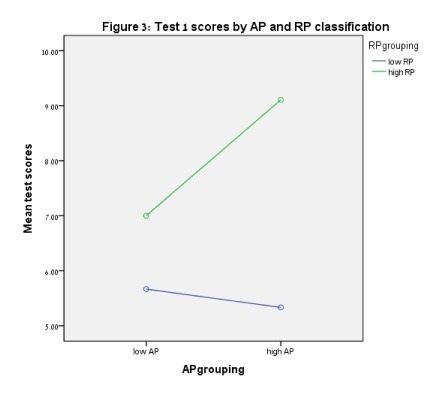
Test 1 scores by AP and RP classification.

**Figure 4.**
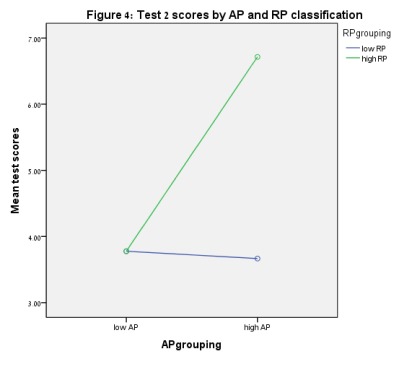
Test 2 scores by AP and RP classification.

**Figure 5.**
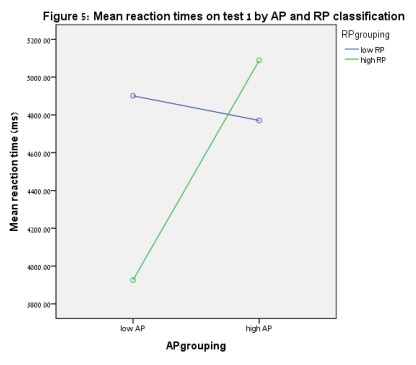
Mean reaction times on Test 1 by AP and RP classification.

**Figure 6.**
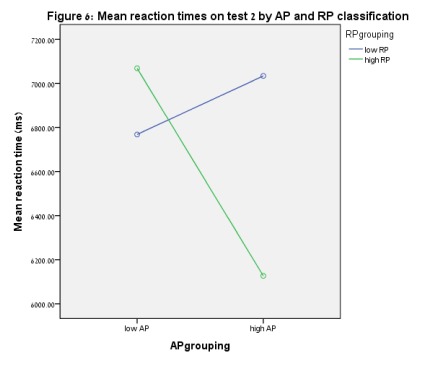
Mean reaction times on Test 2 by AP and RP classification.

In order to test the hypotheses, a multivariate analysis of variance was conducted, with AP (high/low) and RP (high/low) as fixed factors, and Scores and Reaction Times on Tests 1 and 2 as dependent variables. For Test 1 scores, a main effect of RP was found, *F*(1, 69) = 5.9,p = .018. Participants with high RP scored higher (*M* = 8.59, *SD* = 4.03) than participants with low RP (*M* = 5.58, *SD* = 3.69). No main effect for AP was found, and no interactions between AP and RP were found. Figure 3 presents scores for Test 1. For Test 2 scores, a main effect approaching significance was found for AP, *F*(1, 69) = 3.81, *p* = .055. Participants with high AP scored higher (*M* = 5.97, *SD* = 3.42) than participants with low AP (*M* = 3.77, *SD* = 1.92). A main effect for RP was found, *F*(1, 69) = 4.43, *p* = .039. Participants with high RP scored higher (*M* = 6.0, *SD* = 3.41) than participants with low RP (*M* = 3.75, *SD* = 1.91). An interaction between AP and RP was found, *F*(1, 69) = 4.43, *p* = .039. Figure 4 presents scores for Test 2. For Test 1 reaction times, no main effects were found for AP or RP. A significant interaction between AP and RP was found, *F*(1, 69) = 4.16, *p* = .045. Figure 5 presents reaction times for Test 1. No significant main effects or interactions were found for reaction times on Test 2. Figure 6 presents reaction times for Test 2.

In order to find the sources of the interaction effects, one-way ANOVAs were conducted between the four groups for scores on Test 2 and reaction times for Test 1. On Test 2 scores, participants with high AP/ high RP scored significantly higher than all other groups at *p* < .05. An almost significant difference in reaction times on Test 1 was found between low AP/high RP and high AP/high RP at *p* = .054. When reaction times of the low AP/high RP group was compared to all other groups in a univariate analysis of variance with contrasts, a significant difference was found (*t* = -2.23, *p* = .027). Figures 3 to 6 show these results.

## Discussion

The debate regarding the possible advantages or disadvantages of possessing AP centers on the question of whether the local categorical processing of pitch, typical of AP (Schlaug, 2001), is beneficial for processing of global structures in music, dependent on pitch relations or RP (Miyazaki, 2004a; Ward, 1999). The inconsistent results obtained in previous studies comparing performance between AP possessors and non-possessors (Dooley & Deutsch, 2010, 2011; Mito, 2003; Temperley & West Marvin, 2008), and the variance within AP possessors (Bahr et al., 2005; Takeuchi & Hulse, 1993) suggest that different tasks require different types of processing strategies (Miyazaki, 2004a).

In light of this, the present study examined an alternative view to the traditional one of a continuum with AP and RP as its extremes (Levitin & Rogers, 2005). We propose instead that AP and RP are two related but separate abilities, each constituting a continuum. Accordingly, participants were classified by two pre-tests, in which they were asked to name notes with or without interference, into high and low AP and RP. For each pre-test, an AP score (the correct naming of individual pitches) and an RP score (the correct identification of intervals between two consecutively presented pitches) were calculated. The strong correlations found between AP and RP scores on the two pre-tests show that the two abilities are indeed related. However, the validity of the interference procedure in distinguishing between AP and RP was confirmed by *t*-tests comparing scores on the two pre-tests. Interference did not affect AP scores for participants categorized as high AP, but did reduce scores for participants categorized as low AP. Conversely, interference reduced RP scores for participants categorized as high RP, but did not affect scores for participants categorized as low RP. However, as mentioned above, the RP scores were inferred from participants’ responses and not measured directly. In effect, within individuals with AP, there is no direct way to measure RP, since intervals would necessarily be correctly identified, though the strategy used would conceivably be based solely on the identification of single pitches. Nevertheless, the classification of participants by levels of RP may be attempted through more direct RP tasks in future studies.

Participants were divided into four groups, by their AP and RP scores. Raw scores show most participants do not possess very high AP scores, and most participants are either high or low on both AP and RP, resulting in substantially smaller numbers of participants in groups possessing high scores on one ability and low scores on the other than in groups possessing either high or low scores on both abilities. Although ideally a design with more equally distributed numbers of participants in each group would be preferable, the correlation between RP and AP implies that participants high on one ability and low on the other are rare. The *t*-tests described above, as well as the significant results discussed below (in particular, the interaction between AP and RP on reaction times of Test 1) seem to confirm the fact that the two abilities are distinct and continuous. The effect of these two abilities was then tested on an RP and AP task using chords. In Test 1(RP task), participants were asked to identify chord quality, and in Test 2 (AP task), they were asked to name a particular note within a chord. In addition, reaction times were measured. The perception of chords seems an ideal case for studying the difference between global and local processing, since while they are constituted by several individual pitches, they may be perceived as whole single objects (Heaton, 2003). However, the perception of individual chords has rarely been tested in relation to AP. It was hypothesized that high RP would lead to better identification of chord quality, and high AP would slow down reaction times in this task. It was further hypothesized that high AP would lead to better identification of single pitches within a chord and to faster reaction times.

Results generally confirm the hypotheses. In Test 1, the identification of chord quality, a main effect of RP was found, suggesting that this task indeed requires RP. Moreover, the lack of a main effect of AP or an interaction between AP and RP on this task suggests that AP is irrelevant for such a task, which demands global processing. This result is in line with studies showing reduced performance in AP possessors on tasks requiring global processing (Mito, 2003; Miyazaki & Rakowski, 2002), and with studies suggesting that chords tend to be perceived as inseparable wholes (Demany & Ramos, 2005; DeWitt & Crowder, 1987). Although Dooley and Deutsch (2011) found correlations between AP possession and performance on RP tasks, they did not measure RP abilities, and so could not distinguish between AP and RP abilities. Moreover, the task examined in that study was interval identification, and did not use chords. Although it is impossible to ascertain the strategies used by participants to complete the tasks in the present study, the results suggest different strategies in high AP and high RP possessors. Specifically, and most importantly, the interaction between AP and RP on reaction times for this task shows that high AP slows down reaction times. In fact, the fastest reaction times in the present study were found for participants with high RP and low AP, confirming the fact that in spite of unequal numbers of participants in the various groups, RP and AP abilities are distinct. These longer reaction times in high AP possessors are in line with previous studies, which showed that AP possessors reacted more slowly to the identification of a tonic presented after an arpeggiated chord (Wilson et al., 2009) or a transposed melody (Miyazaki, 2004b, Study 1; Miyazaki & Rakowski, 2002). It seems that the automatic nature of the categorical perception of pitch is responsible for this deceleration of response (Wilson et al., 2009). This result is in fact more significant than the correct identification per se, since as mentioned above, if AP possessors do not perceive chord types using RP but through discrete identification of constituting pitches, their raw scores would be equal to participants who use AP strategy. However, since they do not perceive chord type directly, but calculate the type of chords by the individual pitches, their reaction time should be longer. However, as mentioned above, since to our knowledge no previous studies addressed this question in the same manner, additional studies are needed in order to confirm the perception of chord hues as a global structure.

The second test in this study, identifying a single pitch from within a chord, requires analytical processing. This test was more difficult than Test 1, as evidenced by the lower mean scores beyond AP-RP classification and the longer reaction times than in Test 1. A main effect of AP was found for this task, apparently confirming the advantage of AP for local processing. In addition, contrary to the hypothesis, a main effect for RP was also found, suggesting that RP can also be useful in this kind of task. However, a closer look at the interaction between the two shows that performance on this task was significantly higher only when both abilities were high. This suggests that even for an apparently straight-forward AP task, RP is also relevant. Moreover, contrary to the hypothesis, AP and RP abilities did not affect reaction times on this task. This result seems to be in line with Wilson and colleagues’ (2009) results, who found no difference between AP and QAP musicians’ reaction times in correctly identifying single pitches presented after an arpeggiated chord. However, since in that study participants were classified only by their performance on an AP task, it is impossible to ascertain that these results reflect the same effect.

Since to our knowledge the only existing study using chords in relation to AP was conducted by Wilson and colleagues (Wilson et al., 2009), and its methodology was different to the one used here, both in terms of the task and in terms of the classification of participants into groups, it is difficult to generalize the obtained results. However, results of the present study suggest two conclusions. First, conceptualizing AP and RP as different abilities seems to allow a clearer understanding of processing strategies used in various musical tasks. In the present study, the separate consideration of the two groups shows that low AP does not imply high or low RP. In other words, RP is not at the other extreme of AP abilities. Although the two abilities are related, and most participants were either high or low on both, in some cases one is high while the other is low. It is possible that some of the inconsistent results found in previous studies are not simply attributable to different levels of AP, but to a combination of RP and AP levels. In studies using tasks specifically designed to measure RP (Mito, 2003; Miyazaki & Rakowski, 2002; Temperley & West Marvin, 2008; Wilson et al., 2009), categorizing participants not only by their AP abilities, but also by RP, would conceivably render the results more coherent.

The second conclusion regards the processing of global versus local musical factors. At least in the case of isolated chords, the present study suggests that RP is more relevant to the identification of chord quality, and that AP may increase reaction times. In other words, in tasks requiring global processing, the tendency for analytical processing may be irrelevant and slow down performance. The slower reaction times of AP possessors may be likened to the Stroop effect, where a learned response becomes automatic and slows down a more simple reaction. If, as suggested by various studies, global processing normally precedes local processing (Deruelle, Schön, Rondan, & Mancini, 2005; Peretz, 1990; Schiavetto et al., 1999), and AP is a skill acquired in childhood (Pfordresher & Brown, 2009), it may be that this skill, leading to local processing, slows down reaction times in a task requiring global processing.

An interesting question regarding this last point would be whether the tendency to process pitches analytically, evidenced in AP, may be generalized to other domains. Two lines of research point in this direction. The first comes from studies on autistic disorder. Individuals with autism tend to have “weak central coherence”, a tendency to focus on details at the expense of global processing (Happé, 1999). These individuals show better performance in processing local information in music in general, and in pitch identification in particular (Foxton et al., 2003; Mottron, Peretz, & Ménard, 2000) and have a relatively high incidence of AP (Heaton, 2009). The second comes from studies on musicians. A comparison between musicians with and without AP on various cognitive tasks and personality traits found some similarities in AP possessors with characteristics typical of autism spectrum disorders, suggesting that AP is related to more general cognitive and personality features found in autism (Brown et al., 2003). Another study compared musicians and non-musicians and found that musicians’ advantage in local processing is evidenced in their higher performance on visual cognitive tasks (Stoesz, Jakobson, Kilgour, & Lewycky, 2007). The findings of the present study perhaps reflect the flip-side of this phenomenon, that is, a difficulty to integrate local components into a holistic representation in AP possessors. It would be interesting to examine the relationship between AP, RP, and the processing of local versus global factors in musicians in domains other than music.
